# Beyond Exposure to Outdoor Nature: Exploration of the Benefits of a Green Building’s Indoor Environment on Wellbeing

**DOI:** 10.3389/fpsyg.2018.01583

**Published:** 2018-08-30

**Authors:** Bianca C. Dreyer, Simon Coulombe, Stephanie Whitney, Manuel Riemer, Delphine Labbé

**Affiliations:** ^1^Department of Psychology, Faculty of Science, Wilfrid Laurier University, Waterloo, ON, Canada; ^2^Faculty of Environment, Waterloo Institute for Sustainable Energy, University of Waterloo, Waterloo, ON, Canada; ^3^Department of Occupational Science and Occupational Therapy, Faculty of Medicine, The University of British Columbia, Vancouver, BC, Canada

**Keywords:** indoor environmental features, wellbeing, high-performance green buildings, employee mental health, green buildings, nature exposure

## Abstract

Most research exploring the psychological benefits of the natural environment has focused on direct exposure to the outdoors. However, people spend most of their time indoors, particularly in office buildings. Poor employee mental health has become one the most prevalent and costly occupational health issues. The integration of high quality environmental features (e.g., access to sunlight) in green-certified office buildings offers a superior work environment. These nature-based experiences are anticipated to provide beneficial outcomes to wellbeing. This study is the first to empirically investigate these benefits. Participants in a green (LEED gold certified) office building (*N* = 213) in Canada completed an assessment of environmental features, measures of hedonic, eudaimonic and negative wellbeing (NWB) and assessments of psycho-environmental potential, environmental behaviors and social belonging. Linear regression analyses confirmed the benefits of indoor environmental features for all aspects of wellbeing. Multiple regression analyses were conducted to assess the effect of specific indoor environmental features on wellbeing. We explored physical features (e.g., air quality, light), and social features (e.g., privacy), as well as windows to the outside. Results suggest that physical features are important in promoting hedonic wellbeing, while social features prevent NWB. Both features equally predicted eudaimonic wellbeing (EWB). A view to the outside was positively correlated to wellbeing, although it did not uniquely predict it after accounting for other environmental features. Path analyses revealed the importance of person-environment fit, pro-environmental behavior and social belonging in mediating the association of indoor environmental features with hedonic and EWB. The results suggests that, by fostering person-environment fit, pro-environmental behaviors and feeling of community in a high quality setting, green buildings may lead to benefits on an array of wellbeing dimensions. The theoretical and practical implications of these findings are discussed.

## Introduction

There is growing recognition that nature-based experiences provide a range of benefits for physical, mental, and social health (Ulrich, 1984; Ohtsuka et al., 1998; Cimprich and Ronis, 2001; Wu and Lanier, 2003; Loeffler, 2004; Wichrowski et al., 2005; Boniface, 2006; Berman et al., 2008; Park et al., 2010; Van Den Berg and Custers, 2011). Most research exploring the benefits of nature-based experiences emphasize those rare occasions that we actually spend outdoors. However, with urbanization and global migration into urban centers (over 50% of the world’s population is now living in urban areas), exposure to outdoor green spaces is becoming less frequent in people’s everyday life (Zipperer and Pickett, 2012). This trend has serious implications given that exposure to natural environments has been shown to provide long-term improvements in health and wellbeing (Pearson and Craig, 2014). Indeed, data suggest that rapid decreases in exposure to nature are linked to growing mortality and morbidity rates associated with chronic stress and poor mental health (Gärling and Golledge, 1993; Maller et al., 2006; Louv, 2008; Selhub and Logan, 2012). Rapid urbanization also poses a threat to the health of larger natural ecosystems by creating environmental challenges, such as increased pollution, resource depletion, flood risk, elevated temperature and habitat destruction.

Buildings alone account for 40% of global energy use and 38% of global green house gas emissions (UNEP, 2012). A trend to mitigate some of the environmental consequences of buildings is the construction of high-performance green buildings (HPGBs). In addition to being high-performers in regard to environmental impact reduction potential, these buildings also tend to offer opportunities for nature-based experiences within indoor spaces (McSweeney et al., 2014). Design and architectural features of indoor spaces that capture important elements of the natural world, such as contact with sunlight or plants, could potentially harness similar benefits of being in outdoor natural settings. Existing empirical evidence demonstrating this potential is promising but still scarce at this time. Given that individuals in Western societies spend more than 80–90% of their time indoors (Evans and McCoy, 1998; Klepeis et al., 2001; Leech et al., 2002; MacKerron and Mourato, 2013; Setton et al., 2013; Matz et al., 2014), indoor environmental features play an important role in influencing an individual’s mental health and wellbeing, either directly or indirectly (Steele, 1973; Ulrich, 1984; Jutras, 2002). These effects may be particularly prominent in the workplace context, as work-related activities have been identified as a main source of stress (Crompton, 2011). Employee mental health and wellbeing is a growing concern (Dimoff and Kelloway, 2013) and, aside from the time they spend at home, adults spend most of their time working indoors (European Communities, 2004). In addition to benefits associated with sustainability, nature-based features of HPGBs have the potential to play a positive role in the wellbeing of workers. Yet, the nascent literature on these buildings has yet to comprehensively examine this role. Considering the benefits of nature-based experiences and changing urban landscapes, it is imperative potential benefits of nature-based indoor environments and their specific design features are carefully examined. This is the goal of the present study.

### High-Performance Green Buildings and Their Indoor Design

Research on the benefits of exposure to natural environments has often been based on a simplistic “nature” versus “built environment” dichotomy (Pearson and Craig, 2014). Yet, over the last decade there has been a drastic shift in building infrastructures toward those that incorporate nature in the design, which is advocated by several new architectural paradigms such as biophilic design, restorative environmental design, etc. (Kellert, 2005; Hartig et al., 2008). HPGBs are one such incorporation of nature into building design. HPGBs are designed with the goal of a net-zero or net-positive impact on the environment (e.g., producing more or as much energy as is used, and zero non-recyclable waste). Various assessment tools have been designed to evaluate them as “green buildings”; the United States Environmental Protection Agency (2016) provides a commonly cited definition: “Green building is the practice of creating structures and using processes that are environmentally responsible and resource-efficient throughout a building’s life-cycle from design to, construction, operation, maintenance, renovation and deconstruction.” (p. 1).

Key objectives of green building performance include resource use, emissions and waste, as well as inhabitants’ health and comfort issues (Cole, 2012). Thus, in addition to targeting environmental sustainability benefits, such as reducing consumption and adverse environmental impacts, these buildings are also expected to provide a healthy indoor environment to their occupants (Cole, 2012; Xing et al., 2017). To fulfill this requirement, environmental design features, such as provision of natural ventilation, access to sunlight, and use of non-toxic materials, are considered in the development and construction phase of HPGBs (Ravindu et al., 2015). The impact of environmental features of HPGBs, including nature-based ones, has received very limited research attention. According to Heerwagen (2000), the intended impacts of green buildings’ environment can be summarized into two broad domains: strategic performance and human resource development. Strategic performance can be measured with indicators such as resource utilization, productivity, turnover intentions, absenteeism and presenteeism. Research has shown that green buildings tend to affect strategic performance (either self-reported or objectively assessed; Newsham et al., 2017), while other studies have not (see review by Newsham et al., 2013; Thatcher and Milner, 2016). Human resource development focuses on indoor environmental quality of the setting and its influence on human outcomes, such as wellbeing.

To date, studies of green buildings and wellbeing have primarily focused on an examination of the physical aspects (e.g., thermal comfort, air quality, lighting) of indoor quality, but lack attention to the social impact of HPGBs’ indoor design (e.g., noise, privacy, social contact). Overall, perceived or objectively measured physical features of the buildings such as air quality, temperature and controls, have been shown to be better in green buildings, although not consistently across studies and buildings (Thatcher and Milner, 2012, 2014b, 2016; Newsham et al., 2013; Coleman, 2016; Holmgren et al., 2017). Subjective measures typically obtained from post-occupancy evaluation studies suggest that “green” buildings are associated with a high workplace satisfaction (Kim et al., 2005; Kato et al., 2009; Armitage et al., 2011; Thatcher and Milner, 2014b; Pei et al., 2015). For example, occupants of “green” buildings have a greater overall satisfaction with the indoor environment compared to occupants of a conventional building (Kim et al., 2005; Hedge et al., 2014; Liang et al., 2014), even when nearly all physical measurements of the two environments are equal (Newsham et al., 2013; Leder et al., 2016). Yet, there is also some evidence that green buildings under-perform on social measures potentially related to wellbeing, such as noise and privacy (Newsham et al., 2013; Coleman, 2016). The present study aims to take a comprehensive look at green building features, considering the physical and social dimensions of the design. While the positive environmental impacts associated with these kinds of buildings tend to provide motivation for initial investments, it is important to note how they are increasingly appealing to employers who hope to gain additional organizational and human benefits related to employee health, wellbeing, job satisfaction, retention, and productivity (Newsham et al., 2013).

A recent study in the United States found that employees of HPGBs showed 26.4% higher cognitive function scores, 30% fewer sick-building symptoms, and 6.4% higher sleep quality scores than employees in high-performing buildings without green certification (MacNaughton et al., 2017). While this is promising, it is not clear what specific aspects of green buildings contribute to these differences and whether or not green buildings affected the wellbeing of building occupants (MacNaughton et al., 2017). While HPGBs are likely to provide access to nature-based experiences (through features such as windows and natural light) we do not clearly know their impact on wellbeing.

### Wellbeing and the Workplace

“Poor employee mental health has become one of Canada’s most prevalent and costly occupational health issues” (Dimoff and Kelloway, 2013, p. 203). Mental health issues are a leading cause of work losses, in terms of absenteeism, presenteeism and sick leaves (Pinheiro et al., 2017). For example, according to the Conference Board of Canada (2012), the country in which the present study was conducted, negative impacts of mood and anxiety disorders on people’s labor participation can be estimated to 20.7 billion Canadian dollars per year. Consistent with the World Health Organization that defines health as “a state of complete physical, mental and social wellbeing and not merely the absence of disease or infirmity” (World Health Organization, 2006, p. 1), we define wellbeing as a multidimensional construct measured by a constellation of positive and negative indicators. This definition is also based on research from the field of positive psychology, highlighting the importance of measuring both negative wellbeing (NWB) and positive aspects of wellbeing in order to get a full portrait of people’s mental health, as these are distinct dimensions found to be influenced by different factors (Keyes, 2002; Huppert and Whittington, 2003). NWB encompasses symptoms of mental illnesses such as anxiety and depression (Westerhof and Keyes, 2010). In contrast, positive wellbeing is made of hedonic aspects focused on pleasurable emotions, such as satisfaction and happiness, as well as eudaimonic aspects focused on actualizing one’s individual and social potential, for example through growth, purpose in life and flourishing relationships with others and the society (Ryff, 1989; Ryan and Deci, 2001; Westerhof and Keyes, 2010). Recent studies highlight that promoting positive wellbeing and preventing NWB go hand in hand: today’s gains in positive wellbeing decrease the odds of developing mental disorders in the future (Keyes et al., 2010).

Workplaces are recognized as a key setting on which to focus wellbeing promotion efforts, in order to help preventing chronic disorders and disabilities (Oldenburg et al., 2002; Shain and Kramer, 2004). Our research is framed within a prevention/promotion perspective (Cowen, 2000; Nelson and Prilleltensky, 2010). In contrast to post-ailment treatment plans, policymakers’, managers’ and designers’ actions to improve workplace’s indoor environment can have much larger impacts by preventing new cases of mental health disorders (i.e., NWB) in the population, and also by promoting overall flourishing (i.e., positive wellbeing).

### The Role of Indoor Environments on Mental Health and Wellbeing

Outside the home, office employees spend most of their waking time inside the buildings they work (Leech et al., 2002; Schweizer et al., 2007) and many of the day’s most stressful tasks occur at work (Aries et al., 2010). Thus, the indoor environments in which people work are important contributing factors to employees’ wellbeing. A significant proportion of the research examining the benefits of indoor environmental features has focused on access to a window view. This has been examined in various setting such as hospital (Ulrich, 1984), long term care facilities (Kearney and Winterbottom, 2006), and workplace (Aries et al., 2010). Indeed, office workers with a window view looking out to nature have been found to report less stress (Shin, 2007; Lottrup et al., 2013), lower levels of tension and anxiety (Leather et al., 1998; Beute et al., 2011), greater job satisfaction (Kaplan, 1993; Shin, 2007; Lottrup et al., 2015) and greater overall subjective wellbeing (Kaplan, 1993). Kaplan (1993) attributes the effects of a window view to ‘micro-restorative’ experiences. While brief, views of nature through a window provide micro-restorative benefits with significant cumulative influences on wellbeing, Kaplan (1993) argues. Besides a window view, little is known about how other indoor environmental features may influence the mental health and wellbeing of employees. McSweeney et al. (2014) conducted a scoping review of the available literature related to the benefits of exposure to nature while indoors. Most of the reviewed research focused on one-time, short-term exposure to either real or representations (e.g., photographs) of single nature-based items, rather than nature-based features more broadly. Further, 59% of the research reviewed used plants and 37% windows with a nature view as their real nature exposure stimulus. Representations were photographs, paintings or videos of natural landscapes. Yet, none of the studies investigated long-term or continual exposure to these elements in participants’ real work environments or considered the other environmental features present in green office buildings (e.g., noise, privacy), a clear shortcoming to the practical application of the study results (Berto, 2005; Barton and Pretty, 2010; Bratman et al., 2012).

Furthermore, it is important to consider individual differences in how indoor environmental features are perceived. In the post-occupancy study of the Centre for Interactive Research on Sustainability (CIRS), a ground-breaking HPGB in Vancouver, Coleman (2016) found that the experiences of the building assessed in the post-occupancy assessment often diverged from the pre-assessment expectations, which influenced participants’ feelings of satisfaction with the indoor environmental features. These discrepancies as well as preferences for different temperatures, light exposure, levels of privacy and even window view can change the effect of environmental features on wellbeing. In addition, some authors found a preference bias for green buildings (Holmgren et al., 2017). Individuals might thus perceive environmental features of green buildings to be more pleasant than the ones of conventional buildings, even when they are equal. Indeed, to some degree, an individual’s experience with nature and other aspects of HPGBs is likely to be idiosyncratic (Patterson et al., 1998). Thus, in addition to understanding how certain groups of people react and engage with natural-based features, we need to consider individuals’ satisfaction with the environmental features of their own workspaces.

People’s satisfaction with HPGB’s environmental features has, to our knowledge, never been examined with regards to impact on wellbeing as we have comprehensively defined. We have found no study simultaneously providing evidence-based information on the relationships between HPGBs environmental features and each specific aspect of wellbeing (i.e., negative, hedonic, and eudaimonic). A few studies have considered job satisfaction, positive mood, and mental health symptoms (Singh et al., 2010; Newsham et al., 2013; Thatcher and Milner, 2016; Bangwal et al., 2017; Dorsey and Hedge, 2017), in relation to working in a HPGB building. However, these studies have lead to inconsistent results. Furthermore, aspects of eudaimonic wellbeing (EWB) have never been, to our knowledge, empirically examined in HPGBs. Environmental psychology research suggests that the physical environment holds strong potential to enhance individual and social aspects of EWB, for example warmer temperatures are likely to impact one’s psychological growth through actualization of their creative skills (see Heerwagen, 2000), and contact with greenery is likely to promote social ties (Kuo et al., 1998). A detailed portrait of the associations between each HPGBs’ environmental features (physical, and social features and a view outside) and several aspects of wellbeing will be useful to inform the practice of building designers, architects and occupational health professionals. Furthermore, a clear understanding of the mediation mechanisms underlying such associations is needed to guide their work.

### Potential Mediators of the Relationship Between Environmental Features and Wellbeing

#### Psycho-Environmental Potential

People’s appraisal of environmental features has been shown to be better predictors of wellbeing outcomes than objective environmental features (Wright and Kloos, 2007; Weden et al., 2008). Perceived person-environment fit is the process by which people appraise their relationship to space. It refers to “the extent of the possibilities for the individual to fulfill his/her goals and needs through his/her environment” (Moser, 2009, p. 351). It is an important condition to experience wellbeing in a space (Moser, 2009). Applied to the workplace, this principle suggests that satisfaction of one’s needs through the workplace environment is an important process through which workplace design features may influence wellbeing.

The Psycho-Environmental Potential model (Steele, 1973; Jutras, 2002) specifically focuses on the extent to which physical settings support people’s needs. Inspired notably by Maslow’s hierarchy of human needs (Maslow’s, 1943), the model identifies six major environmental needs that are consistent with the larger body of literature on human interactions with space (see a review in Augustin, 2009) and that converge with the literature on important dimensions of wellbeing (Coulombe et al., 2016). (1) *Shelter and security* refers to being protected from natural elements and physical threats, and to spaces experienced as refuges or havens; (2) *Social contact regulation* refers to the capacity of a physical setting to supports privacy as well as contact with others, as desired by occupants; (3) *Symbolic identification* refers to the possibility for the users of a space to transmit their values, goals, preferences, beliefs and social status through the environment; (4) *Task instrumentality* refers to the functionality of the environment, allowing occupants to easily perform their tasks and activities; (5) *Pleasure* refers to feelings of gratification associated with being in a space, for example derived from the presence of natural elements (e.g., light, plants, etc.; Labbé et al., 2017); (6) *Growth* refers to a physical environment that promotes the development of skills, and a sense of competence or self-esteem in the users.

Whether objective physical features of the workplace contribute to the potential of a place to support these six needs is yet to be examined, as the model was developed to focus on people’s subjective appraisal of satisfaction of the needs in the environment (Steele, 1973; Labbé et al., 2016). Initially developed for the workplace context, this comprehensive model has strong roots in the organizational development literature (Steele, 1973), however, to our knowledge, quantitative studies using this framework are rare (except Jutras et al., 2015; Coulombe et al., 2016; Labbé et al., 2016, but in the home environment context). The quantitative relationships of those needs with measures of wellbeing and workplace environmental features have never been examined, least of all in the HPGB context.

#### Environmental Behavior

How individuals act within and toward their environment can also have important implications for wellbeing. One important behavior to examine within the context of green buildings is environmental behavior. Engagement in pro-environmental behavior refers to any act by an individual aimed at reducing one’s overall level of consumption, such as recycling, repairing, sharing and re-using products (Kasser, 2017). Indeed, Kasser (2017) provides an overview of 13 articles across several nations and tens of thousands of participants that have demonstrated positive correlations between pro-environmental behaviors and wellbeing. Using self-determination theory, Kasser (2017) argues that increased engagement in pro-environmental behaviors leads to higher level of wellbeing if engaging in the behaviors helps to satisfy people’s psychological needs for competence, autonomy and relatedness. For example De Young (1996) found that people experience more intrinsic motivation and competence motivation when they engage in conservation behavior and Corral-Verdugo et al. (2016) showed increases in intrinsic motives of autonomy, self-efficacy and satisfaction when engaging in thrifty behavior.

Within HPGBs, employees’ environmental behaviors, such as closing windows, wearing weather appropriate clothing, and turning off screen when not in use, are important determinants of the success of these buildings. Some have argued occupants in green buildings adopt pro-environmental behaviors more than occupants of conventional buildings (Steinberg et al., 2009; Azizi et al., 2015). Wu et al. (2013), for example, examined food disposal habits, and found that students in a sustainable building on campus were more likely to dispose of food in the proper bin as compared to students in a non-sustainable building. The authors propose that being in a sustainable, socio-cultural context, primes individuals to act more sustainably. Indeed, Steinberg et al. (2009) showed occupants were more willing to adopt pro-environmental behaviors as encouraged by the green building certification and Azizi et al. (2015) showed that occupants in LEED certified office buildings were more willing to sacrifice their comfort and adopt more pro-environmental behaviors in comparison with the occupants in conventional buildings. Green buildings can thus be regarded as “teaching tools” for environmental education and encourage “green building literacy” (Cole and Jose Valdebenito, 2013; Cole et al., 2013; Izadpanahi and Tucker, 2015; Cole, 2016).

The potential of HPGBs’ indoor design to promote pro-environmental behaviors in the workplace is likely to indirectly contribute to wellbeing. Thus, we will explore engagement in pro-environmental behaviors as a possible mediation process underlying the association of HPGBs’ environmental features to wellbeing.

#### Social Belonging

The spatial and conceptual structure of an environment also shapes interactions within that space, a process that existing models of group behavior often ignore or oversimplify (e.g., in opinion dynamics models or social network analyses; Baumgaertner et al., 2016). Social belonging, the sense of having positive relationships with others, is a fundamental human need (Baumeister and Leary, 1995; Cacioppo and Patrick, 2008). Abundant research has documented positive associations between social relationships and health and wellbeing (Holt-Lunstad et al., 2010; Nieminen et al., 2010). Social isolation or loneliness can have significant negative consequences on wellbeing (Lyubomirsky et al., 2005) and health (Uchino, 2006; Cohen and Janicki-Deverts, 2009). Thus, it is important to examine the role of social belonging in the relationship between the indoor environmental features and wellbeing.

Some scholars suggest that immersion in nature increases social belonging or sense of community. For example, Weinstein et al. (2009) examined the effects of nature on intrinsic (i.e., goals that in themselves fulfill basic psychological needs such as intimacy and community) and extrinsic aspirations (externally valued goods that are not inherently rewarding, but are sought to derive positive regard or reward such as money). They found across four experiments that compared to participants immersed in non-natural environments, participants immersed in natural environments reported higher valuing of pro-social, intrinsic aspirations, also demonstrating greater connection and focus on others and less valuing of extrinsic aspirations. The authors conclude that nature is likely to bring individuals closer to others, whereas human-made environments encourage more selfish goals. At the community neighborhood level, Sugiyama et al. (2008), found perceived social coherence and local social interaction to be associated with perceived greenness. Public housing residents in architecturally similar high-rise buildings had increased use of common spaces and informal contact with neighbors in buildings with larger presence of trees and grass (Kuo et al., 1998). Natural settings, such as community gardens focus on social ties almost necessarily. Gardeners reported higher levels of contact with friends and less feelings of loneliness than non-gardening neighbors in the same age category (Van Den Berg et al., 2010). As another example, Armstrong (2000) and Leyden (2003) found that urban parks facilitate social networks and the building of community and social contacts, which serve to enhance the safety and wellbeing of communities.

However, much less is known about the potential of indoor environmental features to create a sense of social belonging among building occupants or about the mediating role of social belonging on the relationship between nature and wellbeing. One cross-sectional study found social cohesion mediated the relationship between quantity and quality of streetscape greenery and health and mental health (de Vries et al., 2013). However, sense of community or social support were not mediating the relationship between the quality of public open space and mental health in another study (Francis et al., 2012). Weinstein et al. (2009) call for further research to examine whether an effective design that focuses on incorporation of green spaces may promote stronger community identity and care for others, and thus foster the wellbeing of individuals and groups. Our study is testing this possible pathway within the context of green office buildings.

### Objective and Research Questions

Conducted from a comprehensive perspective, the study aims to explore the associations between HPGB environmental features and the wellbeing of employees. Our study will consider for the first time the associations between several environmental features and aspects of wellbeing, as well as several potential mediating factors underlying these associations.

The specific research questions are:

RQ1: How does satisfaction with building environmental features relate to individuals’ wellbeing?
RQ2: How do physical, social and window environmental features relate to hedonic, eudaimonic and negative aspects of wellbeing?
RQ3: What are the mediating processes by which environmental features are associated to wellbeing, considering psycho-environmental potential, pro-environmental behaviors and social belonging?

## Materials and Methods

### Participants and Study Building

Participants (*N* = 214) were employees of a governmental organization in Canada and consisted of 78 men, 128 women, and 7 non-gender-identified participants. Recruitment involved sending out emails to employees by the research team to inform them of the research. Participants received the option of filling out the self-report survey instrument during the free lunch session or using an online survey link that was send out following the lunch. Participants who participated online received a coffee gift card of $10.

All participants worked in a newly built LEED gold certified office building in the downtown core of a Canadian city. The buildings’ LEED certification is prominently displayed in the lobby and participants are aware of the buildings “green” status. At the time of data collection participants had worked in the building between 1 and 7 months. The majority of the participants hold a professional job (59.7%), followed by a technical job (17.6%), administrative job (13.6%) and managerial job (9%). Most participants are union-staff (71.5%), with some participants being in non-union positions (14.9%), supervisor or program manager positions (12.2%) or directorship (1.4%).

The newly built LEED certified office building is 27-stories high with column free office spaces that can be adapted to fit each teams unique needs. The design is focused on an open office concept that includes closed meeting rooms, open cubicle design, and armchairs or other structures for individual work. The building meets LEED Gold certification standards and also promotes the green leasing initiatives of the BOMA Go Green – BOMA BESt (Building Environment Standards) program. Energy is conserved through a mix of high-efficiency equipment and use of passive design strategies (e.g., optimal building orientation). Gray water is recycled for water closest and landscaping focuses on 100% native or adapted species to eliminate the need for irrigation. High quality materials were used in the construction phase to limit emissions and provide a non-toxic work environment.

This study was carried out in accordance with Wilfrid Laurier University’s Research Ethics Board with written consent from all participants before completing the survey.

### Measures

Identical paper and online self-report survey instruments were used. The online survey was implemented on the Qualtrics survey website. The survey included items on satisfaction with workplace indoor environmental features, wellbeing, potential mediators (psycho-environmental potential, environmental behavior and social belonging) and a section on demographics.

#### Indoor Environmental Features

The environmental features rating (EFR) scale is a modified 16-item assessment of participants’ satisfaction with their indoor environment (Newsham et al., 2013). Participants rated their satisfaction with different environmental features such as “Your access to a view of outside from where you sit,” “Air movement in your work area,” and “Level of visual privacy within your office” using a 7-point Likert scale ranging from 1 (Very dissatisfactory) to 7 (Very Satisfactory). The total score is calculated by averaging all items, where higher numbers represent stronger satisfaction with building environmental features. Cronbach’s alpha indicated good internal consistency for the total EFR scale, α = 0.92. An explorative factor analysis supported three subscales, social features (privacy/enclosure and noise), physical features (light, temperature, and air quality/movement) and a single item factor, view to the outside. Cronbach’s alphas of 0.88 and 0.92, respectively, indicated good internal consistency for the social and physical features subscales. Given the importance placed on windows in previous literature, we decided to investigate it as a separate factor, despite the limitation of a single-item measure. See **Table 1** for a full list of items and item descriptives.

**Table 1:** Environmental features rating (EFR) – items and descriptive statistics.

	*N*	*M*	*SD*
**Physical**			
Amount of lighting on the desktop	212	5.27	1.88
Overall air quality in your work area	210	5.31	1.61
Temperature in your work area	211	3.86	1.81
Amount of light for computer work	212	5.11	1.83
Amount of reflected light or glare on the computer screen	212	4.44	2.02
Air movement in your work area	213	4.80	1.70
Quality of lighting in your work area	213	5.01	1.90
**Social**			
Level of visual privacy within your office	212	3.59	1.96
Amount of noise from other people’s conversations while you are at your workstation	211	3.34	1.85
Size of your personal workspace to accommodate your work, materials, and visitors	212	4.90	1.93
Amount of background noise (i.e., not speech) you hear at your workstation	213	4.10	1.93
Level of privacy for conversation in your office	212	3.17	1.92
Frequency of distractions from other people	212	3.42	1.90
Degree of enclosure of your work area by walls, screens or furniture	213	3.99	1.92
Distance between you and other people you work with	212	4.61	1.87
**View to outside**			
Your access to a view of outside from where you sit	212	5.21	2.05

#### Wellbeing

Participants completed several scales and items assessing various aspects of wellbeing: the Scale of Positive and Negative Experiences (SPANE) (Diener et al., 2010), a general life satisfaction item (Newsham et al., 2013), the Flourishing scale (Diener et al., 2010) and the Patient Health Questionnaire-4 (PHQ4) (Kroenke et al., 2009). The SPANE consists of 12-items, including six items to assess positive feelings and six items to assess negative feelings. Each SPANE item is scored on a scale ranging from 1 (very rarely or never) to 5 (very often or always). Diener et al. (2010) demonstrated that the positive and negative subscales reported excellent internal reliability with α’s = 0.89 and 0.86. General life satisfaction was assessed using a single-item adapted from Newsham et al. (2013). The Flourishing scale (Diener et al., 2010) is an 8-item assessment of EWB with good demonstrated internal reliability, α = 0.88. It includes items such as “I lead a purposeful and meaningful life” and “I actively contribute to the happiness and wellbeing of others,” answered on a scale from 1 (strongly disagree) to 7 (strongly agree). The PHQ4 (Kroenke et al., 2009), a four-item screening measure for anxiety and depression, was used to assess mental illness-related symptoms in the last 2 weeks (e.g., “Feeling nervous, anxious or on edge” and “Little interest or pleasure in doing”), on a scale from 1 (not at all) to 4 (everyday). The scale showed appropriate internal consistency, α = 0.84.

After standardizing all wellbeing items, the positive SPANE subscale and participants’ general life satisfaction score were aggregated to provide an index of hedonic wellbeing. EWB was assessed using the Flourishing Scale. Negative mental health was indexed by aggregating the negative SPANE subscale and the PHQ4.

#### Mediators

The psycho-environmental potential framework was used to develop a 7-item assessment of psycho-environmental potential (α = 0.89). Participants rated the degree to which their workplace’s physical environment for example “feels safe” and “offers opportunities to have positive interactions with others,” on a Likert-scale from 1 (not at all) to 5 (completely). Environmental behavior was assessed using seven items (α = 0.76) of workplace pro-environmental behaviors (Robertson and Barling, 2013) assessing the frequency with which participants perform environmentally friendly behaviors at work such as “turning off the lights when not is use” on a scale from 1 (never) to 5 (always). Focused on participants feelings of belonging to their organization, social belonging was measured using the eight-item Sense of Belonging subscale (α = 0.85) of the psychological ‘sense of community in the workplace’ measurement system developed by Burroughs and Eby (1998). Participants indicated their agreement, from 1 (strongly disagree) to 5 (strongly agree), with items such as “I feel loyal to the people in this organization” and “There is a friendly atmosphere in this organization.”

### Statistical Analysis

Specific analytical strategies and software were employed to examine each research question. The descriptive statistics and regression analyses were conducted using the SPSS statistical software package. Path analyses were conducted using MPlus. First, descriptive statistics were computed to examine means, standard deviations and scale reliability of the study variables. Next, the aim was to investigate the effect of satisfaction with overall environmental features on participants hedonic (HWB) and EWB and NWB (Research Question 1). Three separate logistic regressions were conducted; the first examined HWB, the second EWB and the third NWB as predicted by participants’ EFR. Participants missing more than 80% of the items on any of the variables were excluded from the analyses.

Next, to examine which aspects of satisfaction with environmental features relate to wellbeing variables (Research Question 2) we first computed zero-order correlations between the overall EFR and the three aspects of environmental features (physical, social, window view) and wellbeing (HWB, EWB, and NWB). Next, three separate regressions were employed; entering all distinct elements of environmental features block-wise and regressing the three dependent wellbeing variables on them.

To examine the processes by which environmental features influence HWB, EWB, and NWB, a path analysis mediation model was conducted including psycho-environmental potential, environmental behaviors and social belonging as potential mediators (Research Question 3). To assess the mediation model, several indices of fit were considered, with the following being indicative of a satisfactory fit: Comparative Fit Index (CFI) and Tucker Lewis Index (TLI) ≥ 0,95; Root Mean Square Error of Approximation (RMSEA) ≤ 0,06; Root Mean Square Residual (SRMR) ≤ 0,08 (Hu and Bentler, 1999; Hooper et al., 2008). In case of a non-satisfactory fit, using an iterative process, non-significant pathways were removed and additional relationships between variables were added, based on modification indices provided by the Mplus software, also taken into account theoretical considerations.

## Results

**Table 2** presents descriptive statistics of the overall satisfaction with indoor environmental features and the three subscale scores, the wellbeing variables, and the explored mediators. The quantitative data analyses are reported below following the order of the three research questions. As shown in **Table 2**, percentages of missing values were relatively small (between 0 and 6.5%), except for Environmental Behaviors, which was answered by 64% of participants. This specific variable was only used in the path analysis model tested using the Mplus software, implementing a full information maximum likelihood. It has been shown to be one of the most robust approaches to deal with missing values, and it has the advantage of not requiring to delete cases or to impute values (Schlomer et al., 2010; Newman, 2014).

**Table 2:** Basic descriptive statistics for all variables including Cronbach alpha.

Variable	*N*	*M*	*SD*	*a*
Environmental features total	200	4.38	1.24	0.92
Physical	205	4.83	1.38	0.88
Social	207	3.89	1.53	0.92
View to outside		5.21	2.05	–
HWB	206	-0.0005	0.87	0.89
EWB	214	-0.0086	1.0	0.89
NWB	208	-0.0001	0.92	0.90
Psycho-environmental potential	211	3.45	0.85	0.89
Environmental behavior	137	3.91	0.68	0.76
Social belonging	205	3.92	0.68	0.85

### Environmental Features and Wellbeing

Three simple linear regression analyses were conducted to determine if satisfaction with indoor environmental features predicts participants’ HWB, EWB, and NWB. Results of the first simple linear regression suggests that satisfaction with environmental features is a significant predictor of HWB (β = 0.18), *F*(1,211) = 15.52, *p* < 0.001, with an *R*^2^ of 0.07. Higher satisfaction with the indoor environmental features predicted higher levels of hedonic wellbeing in employees of the green building. Satisfaction with environmental features is also a significant predictor of EWB (β = 0.28), *F*(1,211) = 28.61, *p* < 0.001, with an *R*^2^ of 0.12. That is, employees with greater satisfaction with their indoor environmental features also showed greater levels of EWB. Lastly, NWB was significantly predicted by satisfaction with environmental features (β = -0.19), *F*(1,210) = 14.07, *p* < 0.001, with an *R*^2^ of 0.06. Higher levels of satisfaction with indoor environmental features predicted lower levels of NWB in the employees.

Next we wanted to examine whether all aspects of indoor environmental features relate to wellbeing or whether there are elements that are especially important in predicting the different aspects of wellbeing and mental health.

First, correlations between the overall EFR, the three subscales (physical, social, and window), HWB, EWB, and NWB were examined using Pearson bivariate correlations. The results are shown in **Table 3**. Significant positive correlations were found between HWB, EWB and all measures of satisfaction with environmental features. Likewise, NWB was significantly negatively correlated with all measures of satisfaction with environmental features.

**Table 3:** Pearson correlation coefficients for environmental features and wellbeing.

Measure	1	2	3	4	5	6	7
(1) Environmental features total	–	0.833^∗∗∗^	0.884^∗∗∗^	0.394^∗∗∗^	0.265^∗∗∗^	0.352^∗∗∗^	-0.258^∗∗∗^
(2) Physical	0.833^∗∗∗^	–	0.503^∗∗∗^	0.249^∗∗∗^	0.216^∗∗^	0.276^∗∗∗^	-0.213^∗∗^
(3) Social	0.884^∗∗∗^	0.503^∗∗∗^	–	0.283^∗∗∗^	0.228^∗∗∗^	0.309^∗∗∗^	-0.207^∗∗^
(4) View to outside	0.394^∗∗∗^	0.249^∗∗∗^	0.283^∗∗∗^	–	0.147^∗^	0.192^∗∗^	-0.174^∗^
(5) HWB	0.265^∗∗∗^	0.216^∗∗^	0.228^∗∗∗^	0.147^∗^	–	0.697^∗∗∗^	-0.652^∗∗∗^
(6) EWB	0.352^∗∗∗^	0.276^∗∗∗^	0.309^∗∗∗^	0.192^∗∗^	0.697^∗∗∗^	–	-0.551^∗∗∗^
(7) NWB	-0.258^∗∗∗^	-0.213^∗∗^	-0.207^∗∗^	-0.174^∗^	-0.652^∗∗∗^	-0.551^∗∗∗^	-

^∗∗∗^p < 0.001 (2-tailed), ^∗∗^p < 0.01 (2-tailed), ^∗^p < 0.05 level (2-tailed).

Next, three separate multiple regression analyses were conducted, to assess the unique contribution of each aspect of the environmental features to wellbeing aspects. All environmental features were entered block-wise and regressed on each of the three wellbeing outcomes. The results of the first regression, predicting HWB, indicated that the three aspects of environmental features explained a significant amount of the variance in HWB, *F* = 5.21, *p* < 0.001, *R*^2^ = 0.07. However, physical features (β = 0.13, *p* = 0.11, *ns.*) and view to outside (β = 0.076, *p* = 0.28, *ns.*) did not predict HWB, and social features only marginally explained HWB (β = 0.14, *p* = 0.075). Results of the second regression, predicting EWB, indicated that the three aspects of environmental features on the whole explained a significant amount of the variance in EWB, *F* = 9.71, *p* < 0.001, *R*^2^ = 0.12. Physical features marginally predicted EWB (β = 0.15, *p* = 0.052), social features significantly predicted EWB (β = 0.14, *p* = 0.008), but view to outside did not predict EWB (β = 0.1, *p* = 0.16, *ns.*). Results of the third regression, predicting NWB again indicated that the different aspects of environmental features on the whole explained significant variance in NWB, *F* = 5.43, *p* < 0.001, *R*^2^ = 0.07. Physical features marginally predicted NWB (β = -0.13, *p* = 0.09), however, neither social features (β = -0.12, *p* = 0.13, *ns.*), nor view to outside (β = -0.11, *p* = 0.11, *ns.*) significantly predicted NWB. See **Table 4** for a summary of results.

**Table 4:** Hierarchical Regression analysis of predictors of HWB, EWB, and NWB.

	Regression 1: HWB			Regression 2: EWB			Regression 3: NWB		
Variable	*B*	*SE B*	β	*B*	*SE B*	β	*B*	*SE B*	β
Physical	0.079	0.049	0.125	0.108	0.055	0.148^†^	-0.088	0.052	-0.131^†^
Social	0.08	0.045	0.141^†^	0.135	0.05	0.206^∗∗^	-0.071	0.047	-0.119
View to outside	0.032	0.03	0.076	0.047	0.033	0.096	-0.05	0.032	-0.111
*F*-value		5.212^∗∗^			9.709^∗∗∗^			5.426^∗∗∗^	
*R*-Squared		0.07			0.123			0.073	

*^∗∗∗^p < 0.001, ^∗∗^p < 0.01, ^†^p < 0.1*.

### Exploration of Mediation Processes

**FIGURE 1:**
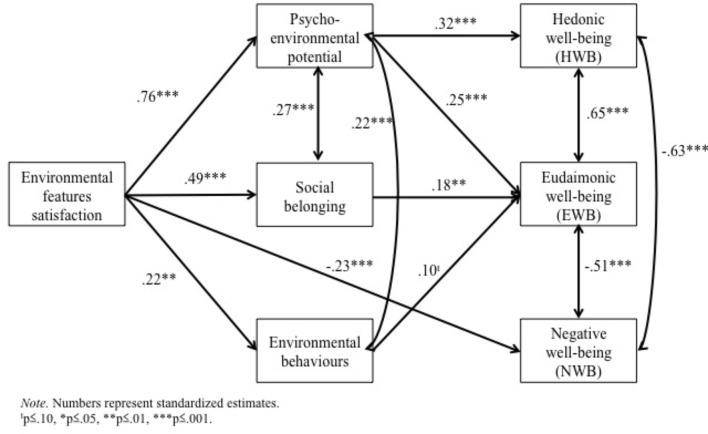
Final path analysis model of the pathways underlying the associations between wellbeing variables and environmental features.

Given that the total score of environmental features is a stronger and more consistent predictor of the wellbeing aspects than the environmental features sub-factors considered individually, we are using the environmental features total score in our path analysis exploring potential mediators. The initial mediation model that we tested included: (a) direct pathways between environmental features and each wellbeing variable (HWB, EWB, and NWB); (b) pathways between environmental features and the three potential mediators (psycho-environmental potential, environmental behavior and social belonging); (c) pathways between these potential mediators and each wellbeing variable; (d) correlations between the three wellbeing outcomes, to acknowledge for their potential interrelationships. This model showed poor fit, χ^2^(3) = 31.05, TLI = 0.67; CFI = 0.96; RMSEA = 0.21 (90% CI [0.15, 0.28]); SRMR = 0.05. The model was improved by iteratively removing non-significant pathways. Based on the modification indices provided by Mplus, correlations were also added between psycho-environmental potential and sense of belonging, and between psycho-environmental potential and environmental behaviors. The final model (see **Figure 1**) indicated a satisfactory fit, χ^2^(8) = 8.97, TLI = 0.996; CFI = 0.998; RMSEA = 0.02 (90% CI [0.00, 0.09]); SRMR = 0.04. The final model included: (a) a direct negative pathway between environmental features and NWB; (b) positive pathways between environmental features and psycho-environmental potential, environmental behavior and social belonging; (c) a positive pathway from psycho-environmental potential to HWB; and (d) a positive pathway from psycho-environmental potential, environmental behavior and social belonging to EWB. Note that all the pathways were significant at *p* < 0.05, except the one from environmental behavior to EWB, which was only marginally significant (*p* = 0.08). Bootstrapped (*n* = 2000 bootstraps) regression estimates were examined to assess the significance of the mediation (i.e., indirect) effects. As shown in **Table 5**, psycho-environmental potential is a complete mediator of the effect of environmental features on HWB, as there is no direct effect between those variables remaining in the final model once the significant indirect effect (i.e., 0 is not included in the confidence interval) through psycho-environmental potential is considered. Environmental features also have significant indirect effects on EWB through psycho-environmental potential and social belonging, and no remaining direct effect was significant in the final model (complete mediation). The effect of environmental features on NWB was not mediated, as there was only a direct pathway between these two variables and no other indirect pathways involved.

**Table 5:** Bootstrap standardized estimates and 95% bias-corrected confidence intervals for direct and indirect effects of environmental features (EFR) on wellbeing (*N* = 213).

	DV: HW			DV: EW			DV: NW		
	*B*	Lo	Hi	*B*	Lo	Hi	*B*	Lo	Hi
Total effect	0.171	0.101	0.241	0.240	0.153	0.327	-0.169	-0.259	-0.077
Indirect effect of IV through…	0.171	0.101	0.241	0.240	0.153	0.327	–	–	–
Psycho-environmental potential	0.171	0.101	0.241	0.153	0.064	0.244	–	–	–
Environmental behaviors	–	–	–	0.018	-0.003	0.050	-	-	-
Sense of belonging	–	–	–	0.069	0.018	0.124	–	–	–
Direct effect	–	–	–	–	–	–	-0.169	-0.259	-0.077

*Results obtained with N = 2000 bootstraps. IV, satisfaction with environmental features (EFR total score). DV, dependent variable*.

## Discussion

This study provides important insights for theory and praxis on indoor environmental features, design and wellbeing by exploring the potential of HPGBs to confer some benefits traditionally associated with outdoor settings (Pearson and Craig, 2014). Urbanization, resource depletion, and lifestyle changes will continue to further limit the possibilities for human contact with outdoor natural environments (Hartig et al., 2014). Previous studies have found, as our study suggests, that indoor nature-based experiences could imitate outdoor natural settings and thus can have positive benefits on wellbeing (Shibata and Suzuki, 2004). Specifically, previous studies have shown that sunlight and a view of nature, potted plants, and photos or illustration of plants or landscapes reduce discomfort (Aries et al., 2010), improve restoration (Kaplan, 2001), increase satisfaction (Ozdemir, 2010), reduce stress (de Kort et al., 2006), improve affect (de Kort et al., 2006), increase wellbeing (Dravigne et al., 2008), and increase positive emotions (Lohr and Pearson-Mims, 2000). Few studies to this point, however, have used holistic conceptualizations of longer-term, multifaceted exposure to a variety of indoor environmental features (social, physical, and view outside), as is the case in green buildings. Doing so, demonstrates the complexity of the relationship between indoor environments designed to be environmentally sustainable (i.e., incorporating a core concern for the natural environment), the wellbeing benefits they may in turn provide and the mediating factors. To our knowledge, this is also the first study to explicitly examine the various factors of wellbeing and demonstrate the importance of the indoor environment in promoting emotional (hedonic) and more profound (eudaimonic) aspects of positive wellbeing and in preventing NWB. The results provide critical insight for linking environmental features of a green office building with the social sustainability of these spaces.

This study’s first research question was to examine whether employees’ overall satisfaction with their indoor environmental features predicts their holistic wellbeing, as assessed by hedonic and eudaimonic positive aspects, as well as negative aspects. The use of satisfaction ratings was thought to be particularly promising, given previous findings about inconsistencies between perceptions of space and objective measures (Coleman, 2016). This study’s findings provided evidence that overall satisfaction with indoor environmental features predicted all aspects of employees’ wellbeing. That is, employees experiencing higher levels of satisfaction with indoor environmental features also reported higher levels of hedonic and EWB and lower levels of NWB. These findings are consistent with other studies showing that one-time, short-term exposure to nature-based stimuli in indoor spaces increase participants’ wellbeing (Kaplan, 2001; McSweeney, 2016). However, beyond previous studies, these findings suggest that one’s workplace environment could significantly affect not just fleeting emotional states, pleasure attainment and emotional pain reduction (hedonic and NWB) but also one’s sense of meaning and self-actualization (EWB). More attention should be paid to the creation of indoor spaces that can provide and enhance these eudaimonic features, which would lead to a more well-rounded assessment of the quality of our indoor spaces.

The second research question explored which aspects of environmental features (physical, social, and view to outside) are associated with different aspects of wellbeing. All aspects of wellbeing were significantly correlated with all aspects of environmental features. Thus, higher satisfaction with social and physical features and with a view outside were related to higher levels of hedonic and EWB and to lower levels of NWB. Past research has paid particular attention to window access as a predictor of wellbeing, affecting both physiological aspects (Ulrich, 1984) and psychological measures of health and wellbeing (Shibata and Suzuki, 2001). While a view to outside was correlated with wellbeing and thus an important factor of wellbeing, this study highlighted that it is not the only aspect of indoor environmental features that needs to be considered. When considered in conjunction with the physical and social aspects of indoor environmental features, view to the outside was not a unique predictor of any aspect of wellbeing. It is possible that because HPGBs place such an emphasis on window access and aim to provide a view to the outside to all employees, that all employees were equally satisfied with their view outside and thus access to a window no longer differentiated between individuals’ assessment of their wellbeing. While the mean satisfaction with the view outside was relatively high (see **Table 2** for the main descriptive statistics of all study variables), there was also considerable variability within the sample. More research needs to explore the unique contribution of window access in green buildings compared to the other indoor environmental features these spaces provide.

Our results further suggest that none of the environmental features are uniquely predictive of hedonic aspects of wellbeing. This suggests that happiness and pleasure-focused aspects of wellbeing are influenced by a holistic perception including several aspects of indoor environmental features, somewhat consistent with a transactional perspective on person-environment relationships (Werner et al., 1992). Previous research on hedonic aspects of wellbeing in green buildings has presented mixed results. Heerwagen (2000) found improvements in emotional and social wellbeing for daytime workers in green buildings but not for shift workers. Singh et al. (2010) found reductions in self-reported depression and stress, but Thatcher and Milner (2012, 2014a,b) found no improvements in mental wellbeing of employees moving into green buildings. Thatcher and Milner (2016) content that it is not “how green you make it – it’s how you make it green” that counts (p. 195). Our mixed findings might be the result of lacking standards for green building design features. As designers and policymakers are become more aware of the importance of designing environments that promote wellbeing and health, guidelines are been developed to help them achieve such purpose. For example, the International WELL Building Institute (2017)^1^ has developed comprehensive standards and a certification process to help guide the work of space designers in order to improve occupants’ wellbeing related to several indoor environmental aspects, for example, air, water, light, fitness, comfort, and mind.

Different indoor environmental features interact with each other, sometimes with unintended consequences. For example, improvements on one dimension of the physical building side (e.g., access to direct sunlight), might inadvertently lead to decreases in social building dimensions (e.g., noise), which was a problem indicated by some participants in qualitative follow-up questions (not described in this paper). However, most previous research on green buildings has not examined these environmental features separately. When studies have used objective measures of physical design features (light, temperature, air) and satisfaction ratings with indoor environmental features concurrently, these measures were not used to predict wellbeing within green buildings, but in “between building” research comparing green building performance to conventional building performance (Newsham et al., 2013).

Lastly, NWB was not uniquely predicted by any one aspect of the indoor environment. Thus, just as was the case with hedonic wellbeing, the relationship between different indoor environmental features and NWB is a complex one. Hartig et al. (2014) note that much research linking nature-based experiences with ill-health focused on intermediate outcomes such as amount of social contact or changes in stress, rather than outcome states of NWB directly, making it difficult to draw conclusions about the relationship between NWB and environmental experiences. In the current context in which employee mental health issues are considered to be a major concern (Dimoff and Kelloway, 2013), looking at the interaction between design and wellbeing and its role in preventing such issues is a crucial endeavor. To our knowledge, this is the first study to separately examine the effect of different features of the indoor environment on the aspects of wellbeing, and it is important to extend our findings to other green buildings and conduct comparative studies of green and conventional buildings with these variables in mind.

The third research question examined some potential mediators of the association between indoor environmental features and wellbeing. The results of our path analysis showed that psycho-environmental potential mediated the relationship between satisfaction with environmental features and the positive aspects of wellbeing. Further, sense of belonging and individual differences in environmental behaviors mediated the relationship between satisfaction with environmental features and EWB. NWB was not mediated by any of our proposed variables. The results concerning sense of social belonging (Staats and Hartig, 2004) and environmental behavior (Khashe et al., 2015) are consistent with previous research. While expected theoretically, the findings concerning psycho-environmental potential add to the heuristic and empirical value of this comprehensive framework (Steele, 1973; Jutras, 2002), never operationalized before in quantitative research, to understand how indoor environments influence wellbeing. Overall these findings show that HPGBs play an important role in fulfilling employees’ psycho-environmental needs, feelings of connectedness, and engagement with environmental behaviors. Since green buildings strive to improve inhabitant health and comfort (Cole, 2012), these results provide additional evidence to their success.

Not all three factors were equally important in explaining the relationship between satisfaction with indoor environmental features and wellbeing. Findings suggest that the person-environment fit, as assessed by the psycho-environmental potential model is a crucial component in promoting positive aspects of wellbeing of employees in HPGBs. Employees satisfaction with indoor environmental features seems to improve their perceived fit with their environment, in turn increasing their hedonic wellbeing. Central to enhancing person-environment fit is creating environments that are adaptable and varied (Mangone et al., 2017). Heerwagen and Diamond (1992) reported that occupants in a field study used repositioning themselves as the most common response (49%) to glare on their computer monitor. Not every office layout will perform equally well for different work-related tasks. Mangone et al. (2017) found that employees preferred natural outdoor spaces for less structured and more abstract activities such as brainstorming, reflection and evaluation, but not for structured and habitual work such as technical/focus and administrative tasks. Our results suggest that, as HPGBs focus on enhancing their integration of nature-based elements into their design, designers and architects needs to remain mindful of individuals’ needs for adaptability and variety to create spaces that facilitate the fulfillment of needs for shelter and security, social contact regulation, symbolic identification, task instrumentality, pleasure and growth. Person-environment-fit did not mediate the relationship between satisfaction with environmental features and NWB, which indicates that while needs fulfillment plays an important role in fostering positive wellbeing, it might be less relevant in preventing negative aspects of wellbeing that arise due to other workplace factors. This aligns with other needs-based frameworks, such as Maslow’s hierarchy of needs (Maslow’s 1943), which focus on human flourishing and achievement of positive wellbeing rather than prevention of NWB. This also adds to the late theoretical work of Lawton suggesting that environmental features promoting positive aspects and the ones reducing negative aspects of wellbeing are not always the same (Lawton, 2001).

Social belonging and environmental behavior were important factors predicting EWB in HPGBs. Previous cross-sectional research has found that social cohesion partially mediated the relationship between neighborhood greenness and mental health (Sugiyama et al., 2008). The present study did not replicate this exact mediation, but rather found social belonging to mediate the relationship between environmental features (including access to window) and growth or self-actualization components of wellbeing. This result is consistent with expectations given that social and community relationships are considered to be key processes involved in EWB (Keyes, 1998; Ryan and Deci, 2001). The fact that environmental behavior was related to EWB is particularly interesting, highlighting how engaging in behaviors at work that protect the environment might contribute to one’ sense of competence, identity, purpose, and self-growth (Ryan and Deci, 2001), beyond the mere emotional aspects (e.g., positive moods). Such findings should be capitalized upon in environmental campaigns, as meaningful and identity-related goals represent an important human motivation factor (Little and Coulombe, 2015).

Further, the role of environmental behaviors in explaining the relationship between satisfaction with environmental features and wellbeing is important to note. An issue of green buildings is the gap in performance in terms of energy, waste and water reductions. Thus, employee environmental behavior is an important determinant of the success of HPGBs to meet reduction targets. So far there are mixed results on the level of employee environmental behavior in green buildings (Day and O’Brien, 2017). This study suggests that satisfaction with indoor environmental features might be an important factor to consider in encouraging employee environmental behaviors, and in turn it predicts employee wellbeing. However, Cole and Jose Valdebenito (2013), Cole (2016), point out that being in a green building is not sufficient to elicit pro-environmental behavior or other positive benefits. Rather than simply being “technically” sustainable, involving the inhabitants in the building’s environmental performance, using the architecture to elicit nature, effective disposal stations, interactive signage throughout building, making environmentally friendly behaviors convenient and engaging, are ways Cole et al. (2013) suggest buildings may motivate their occupants to behave sustainably. In one study, occupants of a green office building engaged in more pro-environmental behaviors than occupants in a conventional building, due to the intervention strategies implemented in the green buildings, such as putting up posters on energy efficiency features of the building (Azizi et al., 2015). These findings are supporting the implementation of some of these strategies in green buildings, including the study building. In constructing a green building it is imperative to keep in mind that physical elements (e.g., including design that promotes contact with nature) alone might not produce the positive benefits one hopes for. Equally important are means of interacting and engaging the building occupants in the goals of the building and ensuring that the building is suitable in fostering their sense of belonging to others, fulfills their main psychological needs of the space and encourages them to act environmentally friendly. Further, consultation with building inhabitants in the construction phase would also enhance satisfaction with the indoor environmental features overall, an aspect that emerges in this study as important for promoting positive wellbeing and preventing NWB.

These results address some questions regarding the basis of benefits provided by indoor environmental features. The present study showed that green building features do not just prevent NWB, as argued by theories such as Attention Restoration Theory (ART) and the psychoevolutionary theory (PET) related to nature-based experiences, but also promote the positive aspects of wellbeing, thus improving the wellbeing of occupants rather than just restoring them to a state of normalcy. These results are promising for the development of HPGBs. The building studied was not uniquely designed to improve employees’ wellbeing; rather it was built to code, following LEED gold certification standards. At time of data collection, no engagement strategies were in place, other than informing employees of the building certification. Thus, it is fair to assume that these results would replicate in other buildings of such kind. If architects and designers become cognizant of the potential of these buildings for health and wellbeing, we would potentially expect the results to be even starker.

One model that already encompasses these considerations is the Regenerative Building Model (du Plessis, 2012). The term “regenerative” describes processes that restore, renew or revitalize their own sources of energy and materials, creating sustainable systems that integrate the needs of society with the integrity of nature (du Plessis, 2012). Regenerativity is a holistic framework that seeks to create systems that are absolutely waste-free, rather than systems that stay within certain pre-determined limits. Thus, regenerative design moves away from a managerial, prescriptive relationship between the human and natural system and moves toward a partnered, co-evolutionary approach that builds rather than diminishes social and natural capitals simultaneously (Cole, 2012). Traditional green buildings often focus on achieving their reduction targets in emissions by focusing on technological solutions (LED, solar power) or behavioral change (recycling campaign). Regenerative design focuses on an integrated approach (Cole et al., 2013) that not only defines the *process* of designing a building but also what constitutes design and who qualifies as the designer. As Reed (2007) describes it, regenerative design shifts the role of the architect/planner/designer away from the expert holding all the knowledge to that of a facilitator of a process of revealing. In a basic sense, it “relies upon every member of the project team sharing a vision of sustainability, and working collaboratively to implement sustainability goals” (Natural Resources Canada, 2015, p. 1). We would expect that the relationship between environmental features and wellbeing would be even stronger in these buildings, given their emphasis on connecting environmental aspects to human wellbeing during the design and construction phase. Further, given the importance of restoration, many regenerative buildings adjust to their natural surroundings, sometimes even mirroring them. For example, buildings might be built around a tree to prevent cutting it down or replicate foliage present in a surrounding forest, as was proposed for the St. Laurent Library in Montreal, Canada (Cucuzzella and Chupin, 2013).

### Limitations

This study’s findings should be interpreted with several limitations in mind. First, unlike research that has used experimental paradigms with limited exposure to nature-based objects, this applied study does not allow for cause-and-effect conclusions. Future research should employ a between-within building design to draw comparative conclusions between buildings (green vs. conventional) as well as important within building conclusions, as highlighted by the current study. Likewise, this study assesses participants’ wellbeing and satisfaction with their environment at one–point in time. We echo researchers such as McSweeney et al. (2014) in calling for longitudinal, pre-post occupancy design research that can more thoroughly investigate the wellbeing benefits of HPGBs following employees from a same organization over time and across buildings (ideally conventional to HPGB). Another option would be for different office spaces within HPGBs to be manipulated to either include more or less nature-based features, thus examining these aspects experimentally. To disentangle the different environmental features, it would also be important to individually manipulate, physical, social and window access while holding other variables (e.g., personality traits potentially influencing people environmental perceptions) constant to further examine the role each of these factors play in wellbeing. As another limitation, in the current measure of satisfaction with environmental features, windows view is the main natural element being measured, and future research would gain in measuring more diverse nature-based aspects such as indoor plants and green walls. Likewise, the applied nature of this study, conducted in partnership with the study setting’s organization, meant that surveys needed to be kept short, which did not allow for the assessment of potential individual difference variables that might contribute to explaining the relationship between indoor environmental features and wellbeing. While this study started to investigate some important environmental features and mediating factors (e.g., person-environment fit, social belonging, and environmental behaviors), it is important to continue to investigate other individual characteristics and their potential influence on the wellbeing benefits of indoor environments. Inclusion of these variables in the regression and path analyses might help explain the remaining variance and further improve the models.

## Conclusion

Despite the limitation of this study, it has provided new insights into the relationship between indoor environmental features and wellbeing. It shows that benefits of nature-based experiences can extend to real-life indoor environmental features of green buildings, adding to previous experimental studies focused on isolated nature-based stimulus in indoor spaces (McSweeney et al., 2014). The benefits of nature-based experiences are well-researched and far-reaching. Our ability to shape our environments allows us to continue to experience these benefits within the changing urban landscape and current society in which several of us spend considerable time working inside; a much-needed task. With increases in workplace mental illness and increasing global stress levels, HPGBs offer a potential avenue to re-focus on health, not just as the absence of disease but the presence of optimal levels of wellbeing. To this end, it is crucial that HPGBs extent their focus beyond limiting reductions and integrate a focus on nature-based elements and wellbeing (e.g., WELL standard, regenerativity), not just calling these buildings ‘green,’ but making them look and feel ‘green’ and good.

Our research suggests that employees’ satisfaction with their indoor environmental features is a crucial factor that may impact their wellbeing. One aspect that reliably increases satisfaction is a sense of control (Taylor and Brown, 1988). This sense of control can be achieved by giving employees control over their environment, e.g., movable furniture, operable windows, different temperature zones, or by increasing employees’ control over the design and construction through early consultation and stakeholder involvement.

Further, in examining how indoor environmental features influence wellbeing, a holistic approach that considers positive and negative aspects needs to be employed. Our built environment holds enormous potential to buffer against poor workplace mental health, one of the largest mental health issues to date. This buffering effect derives from employees’ favorable attitudes toward ‘green’ buildings in general, their satisfaction with specific indoor environmental features and the objective performance of these buildings. It is therefore recommended that the ‘green’ features of buildings are visible (e.g., showing part of the wall insulation through acrylic glass, visible solar panels), communicated (e.g., signage, feedback monitors, email communication) and adaptable (e.g., adjusting shading for better thermal comfort).

As illustrated in the present study, more attention should also be paid to potential mediating factors, such as person-environment fit, environmental behaviors and social belonging. Over and above the technical aspects, buildings are spaces for human life and interactions. In designing indoor spaces, architects, designers and engineers need to consider how humans will co-exist and use the space. The questions we need to ask are: “Do the specific design features fulfill basic psycho-environmental needs?”, “Do these spaces encourage pro-environmental behavior and reward these actions?”, and “Do these spaces facilitate collaboration and belonging?”.

To answer such questions, building certifications need to reach beyond the design phase of the building and start considering the occupancy phase. Paying attention to nature-based elements and other positive environmental features in the design, construction and occupancy of HPGBs may provide an effective means of promoting the wellbeing of the next, increasingly urbanized generation.

## Author Contributions

BD was involved in all phases of the research and led the write up of the manuscript. BD, SC, SW, MR, and DL were involved in the survey design and recruitment. BD and SW were involved in data collection. BD and SC were responsible for data analyses. SC, SW, and MR gave extensive feedback during various phases of the article writing.

## Conflict of Interest Statement

The authors declare that the research was conducted in the absence of any commercial or financial relationships that could be construed as a potential conflict of interest.

## Footnotes

1 https://www.wellcertified.com/en
